# Understanding and Treating Laryngeal Mask Airway-Induced Intraoperative Hiccups

**DOI:** 10.7759/cureus.69782

**Published:** 2024-09-20

**Authors:** Shweta Khatri, Poonam Hannurkar

**Affiliations:** 1 Department of Anesthesiology, Dr. D. Y. Patil Medical College, Hospital and Research Centre, Dr. D. Y. Patil Vidyapeeth (Deemed to be University), Pune, IND

**Keywords:** acute hiccups, atropine sulphate, gastric insufflation, laryngeal mask airways, dexmedetomidine

## Abstract

The laryngeal mask airway (LMA) has gained popularity as an airway management device in the perioperative setting due to its ease of use, reduced invasiveness compared to endotracheal intubation, and lower incidence of complications. However, hiccups, though relatively uncommon, can pose a significant challenge during the perioperative period when an LMA is in place. Abrupt movement because of hiccups can lead to delay in surgery as well as interfere with surgical instrumentation. This discussion presents a case where perioperative hiccups occurred with LMA use and explores the management strategies employed.

## Introduction

Hiccups or singultus are sudden, involuntary contractions of the diaphragm and external intercostal muscles, followed by the abrupt closure of the glottis, producing the distinctive "hic" sound [1]. In recent scenarios, the laryngeal mask airway (LMA) has gained popularity as an airway device for spontaneous and controlled ventilation, as it helps avoid complications associated with endotracheal intubation. Acute hiccup during sedation or general anesthesia after the use of LMA is a minor yet notable complication that can have several disruptive effects. It may disturb the surgical field, making it difficult for the surgeon to perform precise procedures. Additionally, it can interfere with lung ventilation, potentially compromising the patient's oxygenation and ventilation status [2]. This condition can also hinder diagnostic procedures, particularly those requiring steady or controlled conditions, such as imaging or endoscopic examinations. While generally not severe, managing acute hiccups promptly is important to prevent these potential complications. This case suggests that using an LMA device may trigger hiccups, with the incidence of LMA-related hiccups being approximately 1%-14% [3].

## Case presentation

We hereby present a case of a 30-year-old female with a height of 160 cm and 55 kg weight, diagnosed with fibroadenoma in her right breast, scheduled for elective excision surgery under general anesthesia. Classic LMA was decided to be used as an airway adjunct. She had no other significant history, chronic medical conditions, prior surgeries, artificial dentures, or drug allergies.

She was physically fit, with a respiratory rate of 18 breaths/minute, pulse rate of 70 beats/minute, and blood pressure of 110/70 mmHg in the sitting position. On auscultation, breath sounds in both lung fields were normal, and no cardiac murmurs were present. Her airway assessment was Mallampati class I, and she was classified under the American Society of Anesthesiologists (ASA) physical status grade I.

All routine laboratory investigations, including complete blood count, renal function test, liver function test, prothrombin time, and international normalized ratio, were normal with normal ECG and chest X-ray. An informed and written consent was obtained from the patient. The patient was kept nil by mouth for 8 hours preoperatively.

All ASA standard monitors in the operating room were connected, showing a body temperature of 36.8°C, pulse rate of 80 beats/minute, noninvasive blood pressure of 110/70 mmHg, and pulse oximeter saturation of 100%. An intravenous cannula of 20G was taken from the left hand, and 500 mL of Ringer's lactate solution was started.

Premedication with 0.2 mg intravenous glycopyrrolate, 1 mg midazolam, and 2 μg/kg of fentanyl intravenously was done. An oxygen mask was attached at a flow rate of 8 L/minute, and the patient was induced with 2 mg/kg of propofol. A classic LMA of size 3 was inserted, and 20 mL of air was used for inflating the cuff. Anesthesia was maintained with sevoflurane, nitrous oxide, and oxygen. The patient was kept on controlled ventilation, and intraoperative analgesia was achieved with intravenous paracetamol (1 g). The oxygen saturation and pulse rate were continuously monitored. Noninvasive blood pressure was recorded at five-minute intervals during the intraoperative period.

After 20 minutes of induction, the patient suddenly developed hiccups. They continued despite administering 40 mg of propofol in repeated 10 mg boluses. These hiccups disturbed the surgeon's instrumentation during the procedure. Next injection, 0.5 mg atropine was given intravenously but was not effective. Subsequently, 50 μg of dexmedetomidine was administered intravenously over a 10-minute period, which successfully resolved the hiccups. Following the administration of dexmedetomidine, there was a noted decrease in both heart rate and blood pressure, though this did not exceed 20% of the patient's baseline values. The surgery then proceeded without further complications for another 25 minutes.

At the end of surgery, a thorough oral suction was performed, and inhalational agents were discontinued. Once the patient was awake and all airway reflexes were restored, the cuff was deflated, and LMA was removed. She was transferred to the recovery room for further observation. Hiccups did not recur for the next three days while she stayed in the hospital.

## Discussion

Hiccups can significantly disrupt surgery, so addressing them promptly is crucial. The hiccup reflex arc consists of two limbs: afferent and efferent limbs. The afferent limb consists of vagal, sympathetic chains from T6 to T12 and phrenic nerves that travel to the hiccup center located at the posterolateral part of the medulla oblongata. The efferent pathway is through the motor fibers of the phrenic nerve and the accessory nerve to the diaphragm. Any stimulation along the reflex pathway leads to a hiccup. It also increases the risk of aspiration by creating a pressure gradient across the lower esophageal sphincter, enabling reflux.

Various etiologies of intraoperative hiccups are excessive food and carbonated beverages preoperatively [2] and various anesthetic drugs and techniques used during general anesthesia like methohexital, thiopentone, midazolam, and opioids [4,5]. Propofol is also one of the causes of hiccups, which can be treated with intravenous lidocaine [6]. However, since propofol is usually given with lidocaine for ameliorating pain during injection, it is rarely found to be the causative agent (Table 1) [7].

**Table 1:** Management of intraoperative hiccups Pharmacological and nonpharmacological methods used for treating intraoperative hiccups are displayed [8]

Pharmacological management	Nonpharmacological management
Dexmedetomidine	Stomach deairing
Atropine	Lubrication of nasal airway with lignocaine
Midazolam	CPAP of 25-30 H_2_O
Metoclopramide	Acupuncture
Nifedipine	Stellate ganglion block
Proton pump inhibitors	-
Chlorpromazine	-

Bag mask ventilation before induction has pros and cons. On the one hand, it leads to preoxygenation, and on the other, it leads to stomach insufflation, which can cause hiccups due to vagus nerve stimulation. Patients with preexisting conditions like gastroesophageal reflux disease (GERD) and central nervous disorders are also more prone to hiccups.

Patients induced with LMA cause hiccups due to the stimulation of mechanoreceptors. The distal end of a well-seated LMA lies over the proximal esophagus, and the sudden and rapid stretch of mechanoreceptors in the proximal esophagus leads to hiccups. The stimulus for hiccups in intraoperative settings can also be pain at the surgical site and decreased depth of anesthesia [9].

Treatment guidelines are unclear because the reflex arc is incompletely understood, and various possible etiologies exist. The main goal is to disrupt the reflex arc using pharmacological or nonpharmacological methods.

As a lighter plane of anesthesia is one reason for hiccups, we deepened the plane of anesthesia by giving boluses of propofol, but still, hiccups were present.

Acetylcholine is one of the neurotransmitters triggering the reflex arc. Therefore, giving anticholinergics like atropine 0.5 mg intravenously blocks the vagally mediated reflex from LMA insertion and abolishes the efferent arc from the hiccup center [2]. It additionally decreases intraesophageal pressure [3]. In our case, 0.5 mg atropine was unsuccessful in terminating the reflex arc.

In a study by Koteswara and Dubey, dexmedetomidine, a selective alpha-2 adrenergic receptor agonist, was found to abolish hiccups in a similar scenario, implying that sympatholytic drugs abolish intraoperative hiccups [10]. Since atropine was ineffective, we gave dexmedetomidine 1 μg/kg, and the hiccups ceased. There was a slight decrease in heart rate and blood pressure.

Various other modalities can be used in addition to these drugs. For anesthetic agent-induced hiccups, metoclopramide, through its dopamine antagonism and serotonin agonism, seems to be a potent agent for terminating hiccups [8].

Dexmedetomidine offers several advantages over metoclopramide in the management of hiccups. While metoclopramide was traditionally used for treating hiccups due to its dopamine antagonist properties, dexmedetomidine presents unique benefits that may make it preferable.

Sedative, analgesic, and anxiolytic properties

Stimulating presynaptic receptors in sympathetic nerve endings and central postsynaptic receptors inhibits sympathetic activity. Analgesia is achieved by stimulating α2 adrenoreceptors in the spinal cord. This inhibition of sympathetic activity (one of the pathways of the reflex arc in the hiccups pathway), along with analgesic and sedative properties, may have been the reason for suppressing intraoperative hiccups [10].

Reduced risk of adverse neurological effects

Metoclopramide is known to carry risks of extrapyramidal symptoms, especially in young adults who are at higher risk for developing dystonic reactions [11]. Dexmedetomidine, by contrast, lacks these dopamine-blocking effects, which is a significant advantage in younger patients who are more susceptible to these adverse reactions.

GERD patients are, in general, more prone to hiccups, which, in turn, leads to a higher risk of aspiration, so prophylactically proton pump inhibitors should be used. The only Food and Drug Administration-approved treatment is chlorpromazine (25-50 mg) intravenously [12].

Nonpharmacological stomach desiring and nasal airway lubrication with lidocaine lead to pharyngeal stimulation opposite to C2 C3 and terminate hiccups. Lung recruitment, application of 25-30 cm of H_2_O continuous positive airway pressure [2], and suboccipital release technique all reduce pressure on the vagus and interrupt reflex arc [2]. Deliberate hypoventilation, where the end-tidal carbon dioxide is targeted at 48 mmHg, also halts hiccups [13]. Stellate ganglion block is a recent technique for intractable hiccups [14].

## Conclusions

Intraoperative hiccups are a nuisance for both anesthetists and surgeons. Using classic LMA heightens the chance of aspiration, especially in the absence of a nasogastric tube. Hence, strategies to reduce gastric insufflation, early detection of the cause, and effective management using various drug modalities are the key points to prevent any complications.

Numerous interventions have been suggested for managing hiccups during anesthesia. However, perioperative treatment remains largely based on empirical evidence, with no approach being "evidence-based." As a result, no definitive recommendations can be made to treat hiccups. In our case, conventional medications and techniques for multimodal management were ineffective, so dexmedetomidine was administered, successfully controlling the hiccups.
